# Camphorquinone Promotes the Antisenescence Effect via Activating AMPK/SIRT1 in Stem Cells and D-Galactose-Induced Aging Mice

**DOI:** 10.3390/antiox10121916

**Published:** 2021-11-29

**Authors:** Nagarajan Maharajan, Gwang-Won Cho

**Affiliations:** 1 Department of Biology, College of Natural Sciences, Chosun University, Gwangju 501-759, Korea; geneticnaga1990@gmail.com; 2 Department of Life Science, BK21-Plus Research Team for Bioactive Control Technology, Chosun University, Gwangju 61452, Korea

**Keywords:** camphorquinone, oxidative stress, senescence, AMPK, SIRT1, autophagy

## Abstract

Terpenoids are a wide class of secondary metabolites with geroprotective properties that can alter the mechanism of aging and aging-related diseases. Camphorquinone (CQ) is a bicyclic monoterpenoid compound that can be efficiently synthesized through the continuous bromination and oxidation reaction of camphor. The purpose of this study is to investigate the effects of CQ on oxidative-stress-induced senescence and its underlying mechanisms. To generate oxidative stress in human bone marrow mesenchymal stem cells (hBM-MSCs) and mice, we used hydrogen peroxide (200 μM twice) and D-galactose (D-Gal) (150 mg/kg for 10 weeks), respectively. Our findings suggest that CQ potentially reduces senescence in hBM-MSCs and mouse heart tissue. In addition, we found that CQ boosted AMPK/SIRT1 activation and autophagy in both models. These results were subsequently verified in hBM-MSCs using compound C (an AMPK inhibitor) but AMPK inhibition by CC did not significantly reduce the SIRT1 and the autophagy markers. CQ treatment also reduced the gene expression of inflammation markers in D-Gal-induced aging mouse heart tissue. Furthermore, we determined that CQ fits all of the pharmacological parameters using the freely available SwissADME Web tool. Collectively, our findings demonstrate that CQ possesses antisenescence and cardioprotective properties, and that oxidative-stress-induced senescence could be suppressed by AMPK/SIRT1 and autophagy mechanisms.

## 1. Introduction

Cellular senescence is a state of persistent cell cycle arrest that is considered to be a hallmark of aging [1,2,3]. Senescent cells are important in maintaining tissue homeostasis and act as a powerful defense against cancer. Pathological conditions in senescent cells, on the other hand, increase aging and aging-related illnesses [4,5]. Therefore, researchers are searching for new therapeutic medications or targets to eradicate or reduce the negative consequences of these senescent cells while animals age [6,7]. A variety of senescence models, including the oxidative-stress-induced senescence model, have been adopted to better understand the molecular mechanisms underlying senescence. Oxidative stress is a type of stress-induced premature senescence (SIPS) that can be utilized to investigate the mechanism of cellular senescence both in vitro and in vivo [8,9,10,11]. Activation of AMPK, SIRT1, and autophagy mechanisms has been shown to prevent oxidative-stress-induced senescence [11,12,13]. To alleviate oxidative-stress-induced senescence and aging, a novel medication or compound that can activate AMPK, SIRT1, and autophagy is required.

**Figure 1:**
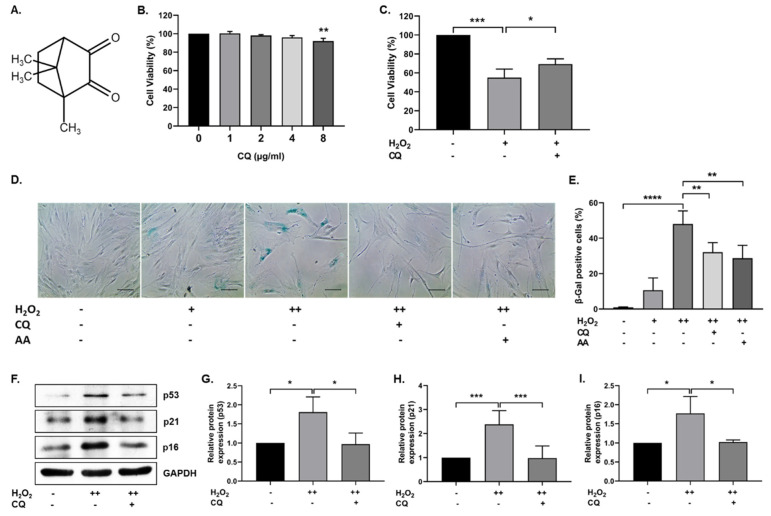
Effect of Camphorquinone (CQ) on oxidative stress-induced senescence. (**A**) Chemical structure of camphorquinone (CQ). (**B**) Effect of CQ on cell viability was measured using the MTT assay (*n* = 3). (**C**) Protective effect of CQ (1 µg/mL) against H_2_O_2_ (0.7 mM) was confirmed via the MTT assay. (**D**) Representative image of SA-β-gal-positive cells in either single- or double-time H_2_O_2_ (200 µM) treatment and (**E**) percentage of SA-β-gal-positive cells in the oxidative-stress-induced senescence model (*n* = 4). (**F**) Representative images from the immunoblot assay against the senescence markers p53, p21, and p16; (**G**–**I**) the expression levels of target proteins were quantified using the ImageJ software. GAPDH was used as an internal control. H_2_O_2_ (+) represents a one-time treatment and H_2_O_2_ (++) represents double-time treatment. The scale bar represents 100 µm. All data are presented as mean ± standard deviation (SD) * *p* < 0.05, ** *p* < 0.01, *** *p* < 0.001 and **** *p* < 0.0001.

Terpenes are a large class of plant secondary metabolites, accounting for about 55% of total secondary metabolites [14]. Terpenoids and their derivatives have a wide range of pharmacological and physiological properties and act as potential geroprotectors [14,15,16]. Based on the number of carbon units, they can be classified as hemiterpenes, monoterpenes, sesquiterpenes, or diterpenes. Monoterpenes are isoprene dimers (C_10_H_16_) that are structurally classified as either acyclic, mono, or bicyclic. Camphor is a bicyclic monoterpene (1,7,7-Trimethylbicyclo[2.2.1]heptan-2-one) found in essential oils from various herbs, including rosemary, lavender, and sage, and is used in traditional Chinese medicine to treat sprains, swelling, and inflammation [17,18]. The antiaging property of camphor has been shown to boost the proliferation and antisenescence effects of human primary dermal fibroblast cells, as well as block UV-induced wrinkles in mouse skin [17]. Camphorquinone (CQ) is a bicyclic monoterpene (1,7,7-Trimethylbicyclo[2.2.1]heptan-2,3-dione) (Figure 1A) that can be efficiently synthesized (~93%) from camphor using a two-step process that includes camphor bromination and the oxidation of 3-brominated camphor, catalyzed by FE-porphyrins, in the presence of air [19,20]. 

The aim of the present study is to investigate the biological benefits of CQ on senescence and aging. The antisenescence efficacy of CQ is evaluated in vitro and in vivo using an oxidative-stress-induced senescence model. The molecular mechanism of CQ and inflammation indicators were investigated in the cardiac tissue of D-Galactose-induced aging mice. In addition, we used the SwissADME Web tool to discuss the pharmacokinetic properties of CQ.

## 2. Materials and Methods

### 2.1. Chemicals and Reagents

Methylthiazolyldiphenyl-tetrazolium bromide (#M2128 MTT) assay kit, hydrogen peroxide (#H1009), ascorbic acid (#A8960), camphorquinone (#124893; purity 97%), and D-galactose (#G0750) were purchased from Sigma-Aldrich (Saint Louis, MO, USA). The SA-β-gal staining assay kit (#9860) was attained from Cell Signaling Technology (Danvers, MA, USA), and Compound C was purchased from Calbiochem (Darmstadt, Germany). Radioimmunoprecipitation (RIPA) lysis buffer (Santa Cruz Biotechnology, Dallas, TX, USA) and Pierce BCA Protein Quantification Kit were purchased from Thermo Fisher Scientific (Rockford, IL, USA). Primary antibodies, including p16 (#sc-1661), p21 (#sc-397), p53 (#sc-6243), MAP LC3α/β (#sc-398822), BECN1 (#sc-48341), SQSTM1 (#sc-48402), SIRT1 (#sc-74465), catalase (#sc-34285), and GAPDH (#sc-365062) were acquired from Santa Cruz Biotechnology, INC. (Dallas, TX, USA). SOD1 (#ADI-SOD-100) and SOD2 (#ab13533) were purchased from Enzo Life Sciences (Lausen, Switzerland) and Abcam (Cambridge, UK), respectively. AMPKα (#5832) and p-AMPK (#2535) were purchased from Cell Signaling Technology (Danvers, MA, USA). The appropriate HRP-conjugated secondary antibodies such as mouse antirabbit (#sc-2357) and mouse antigoat (#sc-2354) antibodies were purchased from Santa Cruz Biotechnology, INC. (Dallas, TX, USA) and the horse antimouse (#7076) antibody was purchased from Cell Signaling Technology (Danvers, MA, USA). The ECL western blotting detection reagents (RPN2209) were purchased from GE Healthcare (Buckinghamshire, UK).

### 2.2. Human Bone Marrow Mesenchymal Stem Cell Culture

Human bone marrow mesenchymal stem cells (hBM-MSCs) were purchased and cultured as described previously [11]. The cells were continuously monitored under a bright field microscope (Nikon Eclipse TS100; Tokyo, Japan) and subcultured as soon as confluence was reached.

### 2.3. Cell Viability Assay

The cytotoxic and protective effects of CQ in hBM-MSCs were determined using the MTT assay kit according to the manufacturer’s instructions. To determine the cytotoxic effect, hBM-MSCs (8 × 10^3^ cells per well) were grown in a 96-well plate. The next day, the cells were treated with different concentrations of CQ (1–8 µg/mL) for 12 h, followed by incubation with the MTT solution for 2 h, and the formazan crystals were dissolved in dimethyl sulfoxide (DMSO). Cell viability was assessed using a spectrophotometer (Multiskan FC, Thermo Fisher Scientific Oy., Vantaa, Finland). For assessing the protective effect of CQ, cells were pre-treated with 1 µg/mL (6.016 µM) CQ for 12 h, followed by incubation with H_2_O_2_ (0.7 mM) for 1 h. Following H_2_O_2_ treatment, cells were treated with the MTT solution for 2 h, and cell viability was evaluated. 

### 2.4. Oxidative-Stress-Induced Senescence

H_2_O_2_ was used to instigate oxidative-stress-induced cell cycle arrest and senescence, as described previously [11]. Briefly, the cells were exposed to 200 µM H_2_O_2_ for 2 h and cultured for 2 days in normal media. For the second round of H_2_O_2_ treatment, the cells were split in a ratio of 1:3, exposed to 200 µM H_2_O_2_ for 2 h, and cultured with either normal media (control), CQ (1 µg/mL), ascorbic acid (500 µM AA; a positive control), or compound C (0.5 µM; AMPK inhibitor) for 72 h. The SA-β-gal assay and western blotting were used to identify cellular senescence, as described below.

### 2.5. Senescence-Associated β-Galactosidase (SA-β-Gal) Staining

Senescence-associated β-galactosidase-positive cells were identified using an SA-β-gal assay kit, as described previously [11]. Briefly, after 72 h of treatment with compounds, cells were washed with phosphate-buffered saline (1X PBS) (Takara Bio Inc., Shiga, Japan) and fixed with 1X fixative for 10–15 min at room temperature. The SA-β-gal staining solution (pH 6.0) was slowly added to the plates, which were then sealed with parafilm and incubated overnight in a dry incubator at 37 °C without CO_2_. The following day, SA-β-Gal-positive cells were observed under a light microscope (Nikon Corporation, Eclipse TS100; Tokyo, Japan) and images were captured using an IMTcam3 cameras (IMT i-Solution Inc., Brook Anco Corporation, Cicero, NY, USA). 

### 2.6. Immunoblotting

Total protein was isolated and quantified as previously described [11]. Briefly, 30–50 µg of protein was separated via SDS–PAGE (6–12%), followed by blotting on a PVDF membrane (GE Healthcare Life science, Catalogue No 10600023, Freiburg, Germany). The membranes were blocked with nonfat dry milk (1%) in TBST for 90 min at room temperature to prevent nonspecific binding of the primary antibodies. Subsequently, the membranes were incubated with the appropriate primary antibodies overnight at 4 °C and then washed with TBST. Next, secondary antibodies conjugated with horseradish peroxidase were added, and an enhanced chemiluminescence detection kit (GE Healthcare) was used to visualize the membranes. Densitometry of the proteins was performed using the ImageJ software (Java 1.6.0_20 (32-bit), National Institutes of Health, MD, USA).

### 2.7. RNA Extraction and qRT-PCR

The RNAisoPlus reagent (Takara Bio Inc., Japan) was used to isolate total RNA from heart tissues; 2.5 µg RNA was then used for cDNA synthesis. The PrimescriptTM II 1st strand cDNA synthesis kit (Takara Bio Inc., Japan) and Power SYBR Green PCRMaster mix (Applied BioSystems, Life Technologies Ltd., Warrington, UK) were used to quantify gene expression levels. The primers were synthesized by GenoTech (Daejeon, Korea) or IDT (Daejeon, Korea). Real-time PCR was performed using the StepOneTM Real-Time PCR system (Applied Bio Systems) (Integrated DNA Technologies, Coralville, IA, USA). The primer sequences used in this study are listed in Table 1.

### 2.8. Animals and Administration of Drugs

Six week-old male mice (C57BL/6; 22 ± 2 g) were obtained from Samtako Bio Korea Co., Ltd. (Osan, Korea) and housed in a pathogen-free animal facility center maintained at 23–25 °C with a 12-h light/dark cycle and free access to drinking water and food. The Institutional Animal Care and Use Committee of Chosun University (CIACUC2020-A0009) approved the animal experiments. The mice were randomly assigned into three groups (*n* = 4) after a week of acclimation: control group (PBS alone), D-Gal model group (administered D-Gal at 150 mg/kg/day), and D-Gal + CQ group (administered D-Gal + CQ at 5 mg/kg/day). The mice were administered either a PBS or D-Gal intraperitoneal injection for 10 weeks or were injected with CQ for 8 weeks starting from the 3rd week of D-Gal injection. Mice were sacrificed at the end of the experiments, and heart tissues were isolated, snap-frozen in liquid nitrogen, and maintained at −80 °C until further use.

### 2.9. SwissADME Prediction

Camphorquinone’s physicochemical, pharmacokinetic, drug-likeness, and medicinal chemistry were predicted using the freely available Web tool SwissADME (Absorption, Distribution, Metabolism, and Excretion) (Swiss Institute of Bioinformatics, Lausanne, Switzerland; http://www.swissadme.ch; accessed on 17 June 2021) and the results were obtained from the same webpage in the form of One-panel-per-molecule output [21].

### 2.10. Statistical Analysis

All data are presented as the mean ± standard deviation of at least three or more biological replicates. The differences between data sets were assessed via Student’s *t*-test and analysis of variance (ANOVA) with Holm–Sidak’s multiple comparison test using GraphPad Prism (GraphPad Software, San Diego, CA). Statistical significance levels are indicated by asterisks as follows: * *p* < 0.05, ** *p* < 0.01, *** *p* < 0.001, and **** *p* < 0.0001.

## 3. Results

### 3.1. CQ Protects hBM-MSCs against Oxidative-Stress-Induced Senescence

To investigate the effect of CQ on cell viability, hBM-MSCs were treated with 0–8 µg/mL (0–48.13 µM) CQ for 12 h, and cell viability was evaluated using the MTT assay. As shown in Figure 1B, there were no significant changes in cell viability up to 4 µg/mL, but viability was reduced at 8 µg/mL. We selected 1 µg/mL concentration (6.016 µM) for further experiments. Next, we evaluated the antioxidant properties of CQ against H_2_O_2_ (0.7 mM). CQ increased the viability of H_2_O_2_-treated hBM-MSCs (Figure 1C). Furthermore, we evaluated the effect of CQ on oxidative-stress-induced senescence, as described previously [11]. As shown in Figure 1D,E, the senescent cells became enlarged, flattened, and highly SA-β-gal-positive (48%) when compared with single-time H_2_O_2_-treated(200 µM) hBM-MSCs (11%). Therefore, we chose double-time H_2_O_2_ (200 µM) treatment for further studies. The number of SA-β-gal-positive cells was considerably reduced upon treatment with either CQ (16%) or ascorbic acid (19%, a positive control). Furthermore, the antisenescence effects of CQ were evaluated on the basis of the expression of well-known senescence markers—including p53, p16, and p21—in our senescence model. As shown in Figure 1F–I, the expression of senescence markers was increased in double-time H_2_O_2_-treated hBM-MSCs, while it was reduced in CQ-treated hBM-MSCs. These results suggest that CQ protects hBM-MSCs from oxidative-stress-induced senescence. 

### 3.2. Activation of AMPK, SIRT1, and Autophagy Reduces Oxidative-Stress-Induced Senescence 

**Figure 2:**
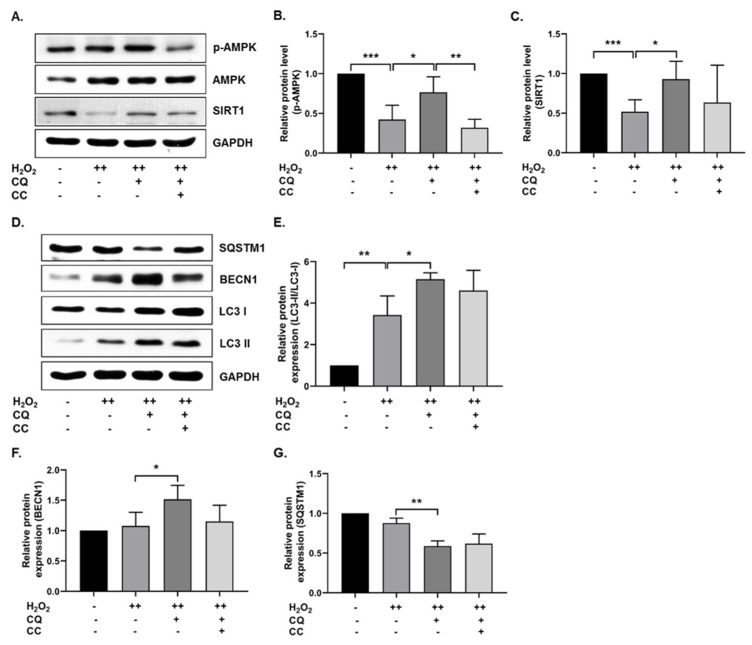
CQ increases autophagy in oxidative stress-induced senescent hBM-MSCs. (**A**–**C**) Representative images from the immunoblot assay against SIRT1, AMPK, and phosphorylated AMPK. (**D**–**G**) Representative images from the immunoblot assay against autophagy markers, including LC3-II/LC3-I ratio, BECN1, and SQSTM1. Compound C (CC)—an AMPK inhibitor—was used. H_2_O_2_ (++) represents double-time treatment. * *p* < 0.05, ** *p* < 0.01, and *** *p* < 0.001.

Previously, it was reported that AMPK-mediated autophagy could protect cells from oxidative-stress-induced senescence [11,22]. In our senescence cell model, we tested whether CQ activated AMPK and autophagy. As shown in Figure 2A–C, SIRT1 and AMPK activation was increased in CQ-treated hBM-MSCs, while AMPK activation was significantly inhibited by compound C (an AMPK inhibitor) but not SIRT1. Next, we evaluated the autophagy mechanisms in our senescence cell model. As shown in Figure 2D–G, CQ increased the LC3-II/LC3-I ratio and BECN1 expression, while it reduced the SQSTM1 expression in our senescence model. Collectively, these data indicate that CQ activates AMPK and SIRT1 mediates autophagy in the oxidative-stress-induced senescent cells, while AMPK inhibition by CC slightly reduced the autophagy markers.

### 3.3. CQ Diminished Cardiac Senescence in D-Gal-Induced Aging Mice

**Figure 3:**
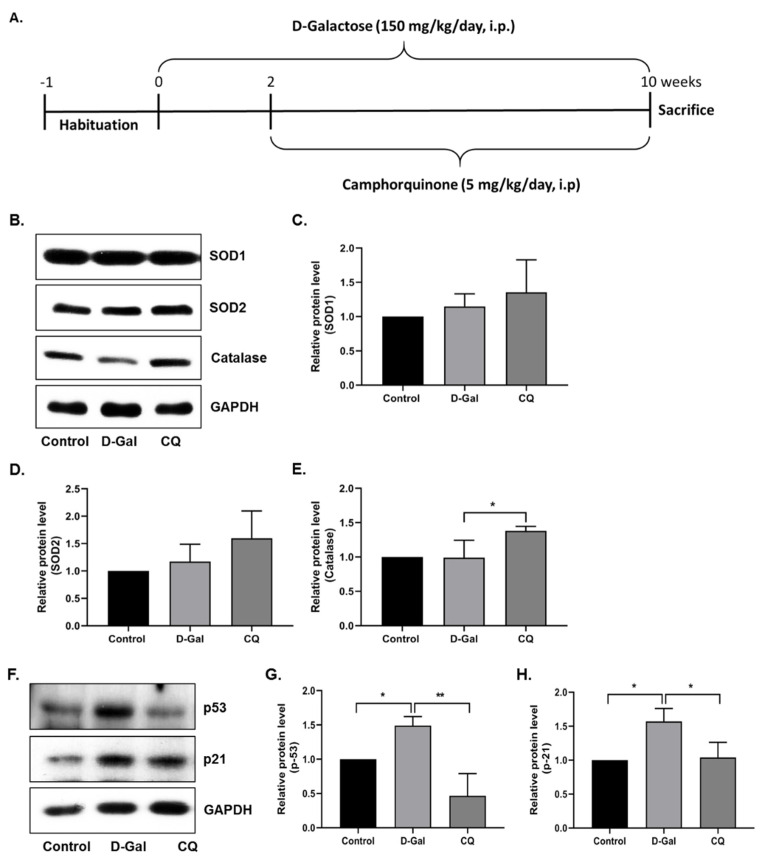
Camphorquinone ameliorates senescence in heart tissue. (**A**) Schematic representation of the experimental plan. (**B**–**E**) Representative images from the immunoblot assay against the antioxidant enzymes SOD1, SOD2, and Catalase from heart tissue. (**F**–**H**) Representative images from the immunoblot assay against p53, p21, and GAPDH from heart tissue. Expression levels of proteins were quantified using the ImageJ software. All data are presented as mean ± SD (*n* = 3–4). * *p* < 0.05; ** *p* < 0.01.

First, we evaluated the antioxidant effect of CQ in D-Gal-induced aging mouse heart tissue. As shown in Figure 3B–E, SOD1 and SOD2 expression levels did not change considerably, while catalase expression increased significantly in the D-Gal-induced aging mouse heart tissue. Next, we evaluated the senescence markers in mouse heart tissue, because long-term administration of D-Gal increased oxidative stress and influenced cardiac senescence in mice [11,23]. As expected, the expression of senescence markers—p53 and p21—was increased in D-Gal-treated mouse heart tissue, while it was considerably reduced in CQ-treated mice (Figure 3F–H). These results suggest that CQ exhibits an antisenescence effect and partially increases antioxidant levels in D-Gal-treated mice.

### 3.4. CQ Activates the AMPK/SIRT1 Autophagy Pathway in D-Gal-Induced Aging Mouse Heart Tissue

**Figure 4:**
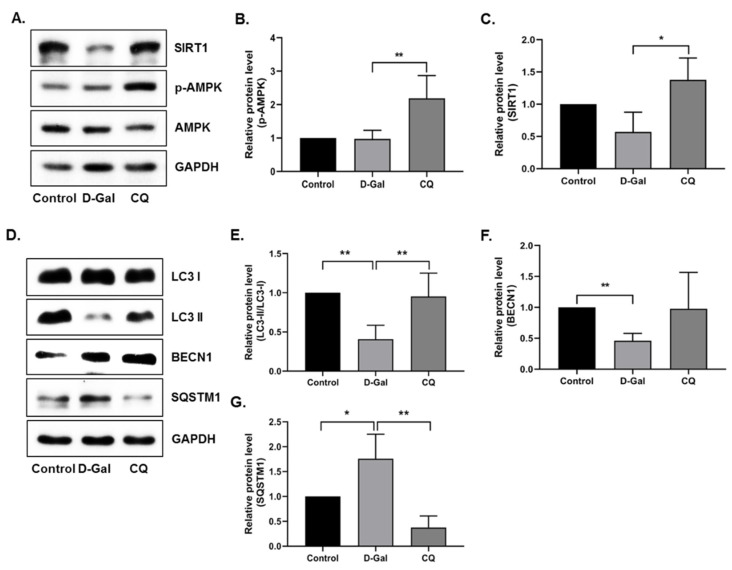
AMPK/SIRT1 activation improved autophagy in D-Gal-induced aging mouse heart tissue. (**A**–**G**) Representative images from the immunoblot assay against LC3-II/LC3-I ratio, BECN1, SQSTM1, and GAPDH from heart tissue. Expression levels of proteins were quantified using the ImageJ software. All data are presented as mean ± SD (*n* = 3). * *p* < 0.05; ** *p* < 0.01.

Next, we examined the activation of AMPK, SIRT1, and autophagy pathways in heart tissue. AMPK and SIRT1 expression was remarkably elevated in CQ-treated mice, but no change or a slight decrease was observed in the D-Gal-induced aging model mice (Figure 4A–C). Furthermore, we examined the autophagy markers in our aging mouse model. As shown in Figure 4D–G, the CQ treatment increased LC3-II/LC3-I ratio and BECN1 expression and decreased SQSTM1 expression compared with that in the D-Gal-treated group. These findings indicate that CQ activates AMPK/SIRT1-mediated autophagy in the cardiac tissues of D-Gal-induced aging mice.

### 3.5. CQ Reduced Cardiac Inflammation in D-Gal-Induced Aging Mice

**Figure 5:**
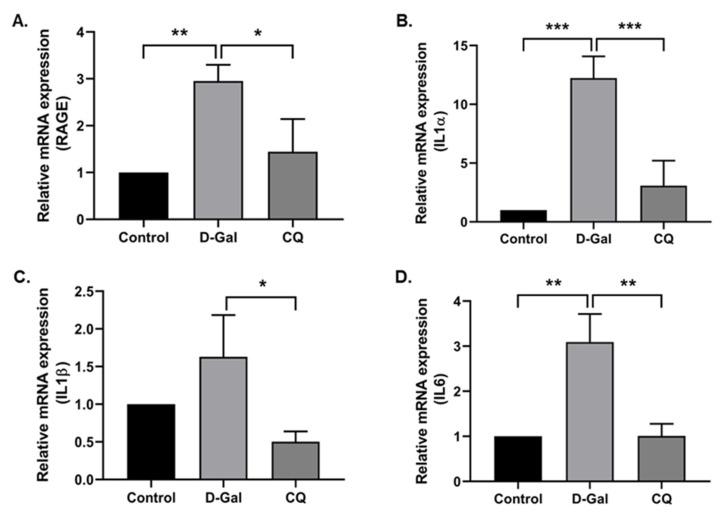
Camphorquinone reduces cardiac inflammation in D-Gal-induced aging mice. RT-PCR for RAGE (**A**) and inflammatory markers (**B**–**D**) in D-Gal-induced aging mouse heart tissue. All data are represented as mean ± SD (*n* = 3–4). * *p* < 0.05; ** *p* < 0.01 and *** *p* < 0.001.

Exogenous administration of D-Gal increases the accumulation of senescent cells in the cardiac tissue and the low-grade inflammation while reducing regenerative capacity, subsequently inducing aging and age-related diseases [10]. With this in mind, we investigated the cardiac inflammation markers in our aging mouse model. Results demonstrated that the D-Gal treatment enhanced aging markers such as the receptor for advanced glycation end product (RAGE) and the levels of inflammation markers (IL1α, IL1β, and IL6) in the D-Gal-induced aging model (Figure 5A–D), whereas CQ treatment markedly reduced the gene expression of these markers.

### 3.6. SwissADME Prediction for CQ

In order to estimate the pharmacokinetic properties of CQ, we performed in silico analysis by the freely available Web tool SwissADME [21]. We obtained following interesting results (Table 2); CQ could be highly absorbable by gastrointestinal tract and may penetrate the blood–brain barrier (BBB), and it is neither a substrate nor an inhibitor of glycoprotein or cytochromes P450 (CYP) isoforms, respectively. Furthermore, CQ falls entirely into the pink zone of the bioavailability radar, which allows for a quick assessment of drug-likeness based on the physicochemical properties such as lipophilicity, size, polarity, solubility, saturation, and flexibility. Most notably, CQ meets the “Lipinski Rule of Five,” which correlates drug-likeness with bioavailability score (0.55) [24].

## 4. Discussion

The pharmacokinetic characteristics such physicochemical, drug-likeness, and medicinal chemistry of CQ encouraged us to explore its biological significance in vitro and in vivo. Therefore, in this preliminary study, we have utilized the oxidative-stress-induced senescence models. We attained the following interesting finding, (i) CQ reduced oxidative-stress-induced senescence in stem cells and cardiac tissue and (ii) CQ activated AMPK/SIRT1-mediated autophagy in both stem cells and D-Gal-induced aging mouse heart tissue.

### 4.1. CQ Improved Oxidative-Stress-Induced Senescence in Stem Cells

Adult stem cells are a promising tool for treating a variety of diseases, particularly in regenerative research. The main disadvantage of stem cells is that they may experience replicative senescence after a long period of replication [25]. Various intrinsic and extrinsic factors can accelerate stem cell aging [26]. Recent studies have shown that low and moderate levels of oxidative stress influence stem cell fate, including quiescence, proliferation, and differentiation. In contrast, excessive ROS levels damage nucleic acids, proteins, and lipids, ultimately causing stem cell senescence and accelerating aging and aging-related diseases [27,28,29]. Antioxidant enzymes, including superoxide dismutase (SOD1 and SOD2), glutathione peroxidase (GPx), and catalase (CAT), play a crucial role in the defense system to regulate cellular redox balance under normal and pathological conditions [30,31]. Defects in the antioxidant system or persistent accumulation of damage lead to the activation of p53/p21, which induces temporal cell cycle arrest by inhibiting cyclin E–Cdk2 [4,32]. Cells may resume normal cell cycle progression if the damage is successfully repaired; otherwise, they may undergo permanent cell cycle arrest or senescence, as well as the release of proinflammatory cytokines [4].

Camphor has been reported to have anti-skin-aging effects as a cosmetic ingredient [17], anti-genotoxic effects at low concentrations [33], and antitumor activity [34]. In this study, we used camphorquinone (CQ), a camphor derivative that is widely used as a photoinitiator, to trigger the polymerization process during dental composite preparation [35]. CQ, at concentrations of 100–2000 µM, has been shown to enhance oxidative stress, inhibit cell cycle progression and differentiation, and induce apoptosis and inflammatory cytokine production in human dental pulp cells [36,37]. In general, several monoterpenes have cytoprotective and antimutagenic properties at low concentrations, whereas the opposite effect is observed at higher concentrations [15]. With this in mind, in this study, low concentrations of CQ were used (Figure 1B; 1 µg/mL, i.e., 6.016 µM), which do not have any toxic effect on cell viability, to investigate the role of CQ in senescence during aging. We employed the oxidative-stress-induced senescence (H_2_O_2_) paradigm in hBM-MSCs to examine the mechanism of stress-induced premature senescence (SIPS) in vitro [11,22]. In the senescence model, our findings implied that CQ reduced the number of SA-β-Gal-positive cells and the expression of senescence markers such as p16, p21, and p53 (Figure 1) [2,38]. Further, we investigated AMPK-mediated autophagy activation as a preventive mechanism that might diminish oxidative-stress-induced senescence [11,22]. It has been proposed that AMPK activation induces autophagosome formation through Unc-51-like autophagy activating kinase (ULK1), while activation of both AMPK and SIRT1 upregulates the expression of autophagy-related genes (Atgs) through FOXO and PGC1α and downregulates mTORC1 [39]. Under caloric restriction conditions, reciprocal activation of the energy sensor molecules AMPK and SIRT1 is thought to inhibit mTOR and enhance autophagy activation [40,41]. Activation of the AMPK/SIRT1-FOXA3-Beclin1 pathway has been shown to not only activate autophagy but also prevent senescence and promote the proliferation of human umbilical vein endothelial cells [42]. Therefore, we analyzed AMPK/SIRT1 and autophagy in our senescence model. We found that activation of AMPK/SIRT1 and autophagy markers was increased upon treatment with CQ in our senescence model, while inhibition of AMPK slightly lowered the autophagy activations (Figure 2). SIRT1 activation, on the other hand, activates autophagy directly [43]. Therefore, activation of AMPK and SIRT1 may regulate the autophagy mechanism. However, in this study, we only utilized AMPK inhibitor but not SIRT1 inhibitor, which could explain why autophagy was not significantly lowered. It should be noted that SIRT1 activation might enhance autophagy and prevent apoptosis partly by deacetylation and degradation of p53 under oxidative stress conditions [44]. This indicates that SIRT1 activation increases autophagy and reduces p53-p21-mediated senescence. Overall, these findings suggest that CQ activates AMPK/SIRT1-mediated autophagy and reduces oxidative-stress-induced premature senescence in stem cells.

### 4.2. CQ Reduced the Senescence of D-Gal-Induced Aging Mouse Heart Tissue

To better understand the action mechanism of CQ in vivo, we used a D-galactose (D-Gal)-induced aging mouse model [10,11,45]. D-Gal is a monosaccharide molecule found in dairy products, peanuts, honey, cherries, and kiwi fruit. For healthy adults, the daily limit for D-galactose is 50 g. D-Gal is metabolized by galactose-metabolizing enzymes, such as galactose mutarotase, galactokinase, galactose1-phosphate uridyltransferase, and UDP-galactose epimerase. Excessive accumulation of D-Gal or alterations in the metabolizing enzymes might have a negative impact on the organism, leading to the onset of aging and age-related disorders [10,45,46]. Herein, we used D-Gal-induced aging mouse models to investigate the effect of CQ on cardiac aging and senescence.

First, we examined the antioxidant effect of CQ on the D-Gal-induced aging of mouse heart tissue. D-Gal can be oxidized by galactose oxidase to form hydrogen peroxide and impair redox homeostasis, resulting in increased oxidative stress and inflammation in the brain and heart [10,45]. We found that CQ significantly increased the levels of the antioxidant enzyme catalase in heart tissue; however, no changes were observed in SOD1 and SOD2 levels (Figure 3). One probable mechanism is that a high quantity of D-Gal can be oxidized by galactose oxidase to produce H_2_O_2_, which can subsequently be reduced or detoxified by GPx and catalase [10,47]; however, SOD converts superoxide radicals into H_2_O_2_ [47]. In this study, as well as our unpublished data, we found that CQ can activate the SIRT1. It also been proposed that activation of SIRT1 upregulates the antioxidant enzymes SOD2 and catalase via deacetylation of FOXO4 and, as a result, inhibits ROS production [48]. Therefore, activation of SIRT1 by CQ is one of the possible reasons why catalase was increased in our study. However, we could not see any changes in the catalase expression in D-Gal treated mice compared with the control group. Next, we evaluated senescence markers in our aging mouse model. Long-term administration of D-Gal causes cardiac dysfunction in heart tissues by increasing cardiac senescence, oxidative stress, inflammation, apoptosis, and decreasing the levels of antioxidants as well as altering calcium homeostasis [10,11]. We found that the levels of senescence markers, including p53 and p21, were significantly reduced in the aging mouse model upon CQ administration (Figure 3). We previously observed that AMPK-mediated autophagy could reduce cardiac senescence in D-Gal-induced aging mice [11]. In the present study, we found that CQ activated AMPK/SIRT1 and autophagy in our mouse model (Figure 4). Furthermore, excessive accumulation of D-Gal can increase ROS production via the Maillard reaction, which converts D-Gal to advanced glycation end products (AGEs). The AGE molecules produced can bind to a receptor called receptor for advanced glycation end product (RAGE), which can enhance ROS production via NADPH oxidase. NADPH increased the activity of p38 MAP kinase, thereby causing NF-kB to translocate to the nucleus, leading to an increase in the expression of senescence-associated secretory phenotype (SASP) including growth factors, transcription factors, and proinflammatory cytokines such as IL-1α, IL-1β, IL-6, and IL-8 [10,49,50]. Chronic activation of these SASP can also cause neighboring cells to produce more SASP into the surrounding environment. In a D-Gal-induced aging mouse model, we found that CQ remarkably reduced heart aging and inflammatory marker levels (Figure 5).

## 5. Conclusions

**Figure 6:**
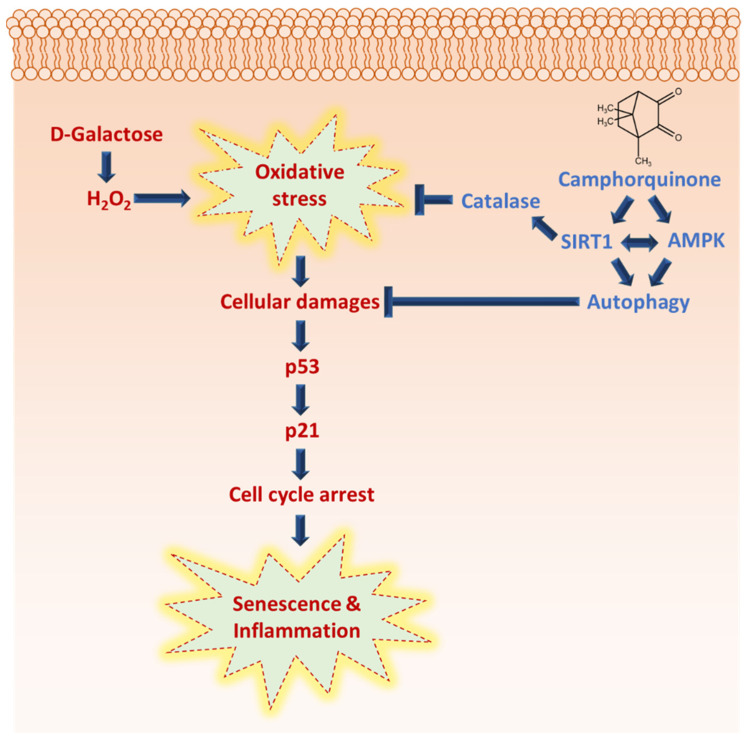
Camphorquinone inhibits oxidative-stress-induced senescence and inflammation via activating AMPK/SIRT1 and autophagy, and SIRT1 activation may regulate oxidative stress by activating antioxidant enzyme catalase.

CQ has been predicted as a pharmacologically active molecule having a wide range of properties, including drug-likeness and bioavailability. This is the first report in which CQ has been shown to diminish senescence and activate the antiaging molecular markers AMPK and SIRT1, the autophagy mechanism, and reduce cardiac tissue inflammation during oxidative stress (Figure 6). However, various kinases, including CaMKKβ and LKB1, as well as metabolic sensors, such as AMP and NAD^+^, can activate the AMPK/SIRT1 pathway. Therefore, it is crucial to determine how CQ activates the AMPK/SIRT1 pathway.

## Figures and Tables

**Table 1:** List of qRT-PCR gene primer sequence.

Target Gene	Gene Accession Number	Sequence (5′→3′)
β-Actin forward	>NM_007393.5	CCACCATGTACCCAGGCATT
β-Actin reverse		CGGACTCATCGTACTCCTGC
Ager forward	>NM_007425.3	AGGTGGGGACATGTGTGTC
Ager reverse		TCTCAGGGTGTCTCCTGGTC
Il1α forward	>NM_010554.4	CCACCAAAGAACAAAGTCGGG
Il1α reverse		CAGACTGTCAGCACTTCCCAA
Il1β forward	>NM_008361.4	AAGAGCCCATCCTCTGTGACT
Il1β reverse		GGAGCCTGTAGTGCAGTTGT
Il6 forward	>NM_031168.2	AGACAAAGCCAGAGTCCTTCAG
Il6 reverse		GAGCATTGGAAATTGGGGTAGG

**Table 2:** SwissADME and physicochemical properties of camphorquinone.

**Molecule**	**Structure** 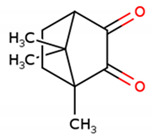	**Bioavailability Radar (For Drug-Likeness)** 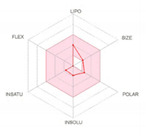
	**Name**	**Camphorquinone**
	**Canonical SMILES**	**CC1(C2CCC1(C(=O)C2=O)C)C**
Physicochemical Properties	Formula	C10H14O2
	Molecular weight	166.22 g/mol
	Number of heavy atoms	12
	Number of aromatic heavy atoms	0
	Fraction Csp3	0.8
	Number of rotatable bonds	0
	Number of H-bond acceptors	2
	Number of H-bond donors	0
	Molar Refractivity	45.84
	Topological polar surface area (TPSA)	34.14 Å^2^
Lipophilicity	Log *P*_o/w_ (iLOGP)	1.55
	Log *P*_o/w_ (XLOGP3)	1.52
	Log *P*_o/w_ (WLOGP)	1.58
	Log *P*_o/w_ (MLOGP)	1.27
	Log *P*_o/w_ (SILICOS-IT)	2.53
	Consensus Log *P*_o/w_	1.69
Water Solubility	Log *S* (ESOL)	−1.83 (Very soluble)
	Log *S* (Ali)	−1.85 (Very soluble)
	Log *S* (SILICOS-IT)	−2.50 (Soluble)
Pharmacokinetics	GI absorption	High
	BBB permeant	Yes
	P-gp substrate	No
	CYP1A2 inhibitor	No
	CYP2C19 inhibitor	No
	CYP2C9 inhibitor	No
	CYP2D6 inhibitor	No
	CYP3A4 inhibitor	No
	Log K_p_ (skin permeation)	−6.23 cm/s
Drug-likeness	Lipinski	Yes; 0 violation
	Ghose	Yes
	Veber	Yes
	Egan	Yes
	Muegge	No. 1 violation: MW < 200
	Bioavailability Score	0.55
Medicinal Chemistry	PAINS	1 alert: imine_one_A
	Brenk	1 alert: diketo_group
	Lead-likeness	No. 1 violation: MW < 250
	Synthetic accessibility	3.37

## Data Availability

The data presented in this study are available in the article.
